# Inequities in home care use among older Canadian adults: Are they corrected by public funding?

**DOI:** 10.1371/journal.pone.0280961

**Published:** 2023-02-02

**Authors:** Afshin Vafaei, Ricardo Rodrigues, Stefania Ilinca, Stefan Fors, Selma Kadi, Eszter Zolyomi, Susan P. Phillips

**Affiliations:** 1 Department of Family Medicine, Queen’s University, Kingston, Ontario, Canada; 2 Department of Public Health Sciences, Queen’s University, Kingston, Ontario, Canada; 3 ISEG Lisbon School of Economics and Management, University of Lisbon, Lisbon, Portugal; 4 SOCIUS, Research Centre in Economic and Organizational Sociology, Lisbon, Portugal; 5 European Centre for Social Welfare Policy and Research, Vienna, Austria; 6 Aging Research Center, Karolinska Institutet & Stockholm University, Stockholm, Sweden; 7 Centre for Epidemiology and Community Medicine, Region Stockholm, Stockholm, Sweden; Emory University, School of Public Health, UNITED STATES

## Abstract

**Background:**

Although care use should parallel needs, enabling and predisposing circumstances including the socio-demographic inequities of socioeconomic status (SES), gender, or isolation often intervene to diminish care. We examine whether availability of state-funded medical and support services at home can rebalance these individual and social inequities, and do this by identifying if and how intersecting social identities predict homecare use among older Canadian adults.

**Methods:**

Using the Canadian Longitudinal Study on Aging (CLSA) of 30,097 community-dwelling adults aged 45 to 85, we performed recursive partitioning regression tree analysis using Chi-Squared automatic interaction detection (CHAID). Combinations of individual and social characteristics including sociodemographic, family-related, physical and psychological measures and contextual indicators of material and social deprivation were explored as possible predictors of formal and informal care use.

**Results:**

Diminished function i.e. increased need, indicated by Activities of Daily Living, was most strongly aligned with formal care use while age, living arrangement, having no partner, depression, self-rated health and chronic medical conditions playing a lesser role in the pathway to use. Notably, sex/gender, were not determinants. Characteristics aligned with informal care were first—need, then country of birth and years since immigration. Both ‘trees’ showed high validity with low risk of misclassification (4.6% and 10.8% for formal and informal care, respectively).

**Conclusions:**

Although often considered marginalised, women, immigrants, or those of lower SES utilised formal care equitably. Formal care was also differentially available to those without the financial or human resources to receive informal care. Need, primarily medical but also arising from living arrangement, rather than SES or gender predicted formal care, indicating that universal government-funded services may rebalance social and individual inequities in formal care use.

## Background

Access to formal homecare tends to align with medical need, but vary with socio-demographics such as income, gender, widowhood, or living arrangement, even when medical need is comparable [1]. In theory, universally available public funding could eliminate socially mediated care inequities in older adults’ access to and use of homecare. This has, however, seldom been directly tested [2]. Testing matters for several reasons. Social circumstances and their sequelae are among the strongest known determinants of health but are also perplexingly resistant to change. Evidence that government-funded universal medical care could rebalance the health impact of social inequities would make a persuasive case for such funding. We will look at whether intersecting social characteristics that are known to undermine use of formal homecare persist in a setting (Canada) with longstanding government funded medical and formal homecare systems.

### Medicare in Canada

A guiding principle of the Canadian universal care system (subsequently referred to as Medicare) is that access to all essential medical care should be unrelated to income, age, sex, location, or other sociodemographic characteristics. However, because of perceived gaps between essential formal homecare paid for by the state, and optimal care, even in Canada accessing a breadth of formal care may confer disproportionate financial burdens upon those with lower incomes [3]. In the Canadian context formal homecare refers to clinical and support services arising from medical need and provided to a person living in the community (and not in an institutional care setting) by a paid worker rather than a family member or a friend. In 2015–16, 91% of in-home nursing care involved no payment by the recipient. However this was true for only 44% of support services [4]. Further, 64.6% of those receiving formal home care had the costs paid for entirely by government as did 36.5% of the recipients of support services [4]. It should be noted that this represents the national average and does not acknowledge variations across provincial jurisdictions. Funding for informal care is not built into the Canadian system (i.e. Canadians receive no direct government payments to provide care to family or friends), making it reasonable to expect that those with limited human or financial resources might require and use formal government-funded care more than would others who, for example, are able to offset a friend’s time commitment to provide informal home care with material compensation. Having access to informal or self-funded care might, therefore, interact with, and diminish the need for formal care and, in so doing, alter the association between social characteristics and formal care receiving. For example, women who lack informal caregivers might appear to utilise more formal care if, as should be the case in countries with Medicare (e.g. Canada), such care can be accessed without personal financial burden.

### Care needs of older adults

As populations age the prevalence of chronic diseases and functional limitations increases, precipitating greater need for homecare [5]. Generally, these needs arise from limitations in function, Activities of Daily Living (ADL) or Instrumental Activities of Daily Living (IADL), and with multimorbidity [6]. However, patterns of care use seem to also be related to the complex interplay of individual characteristics, social networks, and contextual/policy level circumstances. Theoretical models referred to in the literature tend to describe patterns of care use well, but not those characteristics that lead to use. Understanding these possible predictors is a necessary first step in modifying social and healthcare systems to better address the needs of aging populations.

#### Defining formal and informal care

There are no standard definitions of formal and informal care. In general, formal care refers to ‘paid’ services based on or arising from medical need, whereas care delivered by family, relatives, friends, neighbours, or community members is considered to be informal care [7]. Informal and formal care often address different needs and are likely inversely related, with access to the former diminishing the need for the latter [8, 9]. Informal and formal care use will be influenced differently by enabling and predisposing factors (see below for more information on these terms). For example, in settings without state funded care, persons of higher socio-economic status (SES) and greater income are more able to pay for formal services while those of lower SES may need to cobble together informal, cost-free supports [10].

#### Need for, and use of care

Care use can be conceptualised using three dimensions: need for care, predisposing characteristics, and enabling circumstances [11]. Need arising from functional, physical and cognitive health limitations represents the most immediate reason for seeking care [12–14]. Individual-level factors such as age, sex, and health behaviours can predispose to functional limitations that trigger need. Enablers tend to be structural or contextual, and shape care access and use. They include income [10, 15, 16], availability of health insurance, local policies [17, 18], and social support networks [18, 19]. Enabling and predisposing factors sometimes overlap. For instance, gender can be both. Women, in general, have more established social networks that can *enable* informal care. On the other hand, older women more often live alone with no household informal caregiver [20, 21]. In addition, female sex is a recognized risk factor for a variety of chronic diseases and functional deficits that precipitate a need for care [22]. Economic considerations and cultural norms of women’s lower SES relative to men and of men’s stereotypic stoic behaviour add more complexity to gendered patterns of care receiving. When determining the relationship between social characteristics and use of formal care it is therefore important to consider not only single determinants such as sex, income, or living situation, but also the interplay or intersections among these as they shape need, enabling and predisposing dimensions of care.

#### Examining intersectionality and care

With roots in the study of social inequalities and, particularly, gender inequity [23, 24] intersectionality theory assumes that membership in marginalized social groups generates interlocking rather than additive or multiplicative systems of oppression and harm [25]. These “social locations” defined by interconnections or intersections of various social factors create both power and marginalization [24]. Numerous methods for studying intersectionality exist although, as would be expected with a novel framework, none has yet emerged as ‘the best’ [26]. One of these, recursive partitioning, groups individuals using stepwise division of social locations [26–28]. Its output is a graphical ‘tree’ that illustrates how combinations of factors defining homogenous subgroups contribute to the probability of the outcome of interest. Of particular relevance to our research, recursive partitioning allows for identification of subgroups (i.e. those with combinations of characteristics) at higher risk within larger groups that are deemed, overall, to be of low risk for the outcome. This contrasts with traditional regression methods that quantify subgroups at ‘risk’ specifically within the high-risk group. To illustrate this—utilising recursive partitioning one could identify which characteristics precipitate care among a low risk group with no limitation in ADLs.

As stated previously, access to formal home care should align with need arising from cognitive impairment, mobility decline or multimorbidity [7, 29]. Research has also identified disparities in socio-demographics such as gender, widowhood, living arrangement and income that interact with medical circumstances to alter need for care [1]. Specifically, greater need has been documented among women [13, 30], those with lower levels of education [31], and racialized populations [32]. Few, however, have considered intersecting characteristics that predict access to and use of that care. None has examined whether availability of government funded, universal access to medically necessary formal care can correct for the impact of individual or intersecting socio-demographic inequities. Guided by an intersectionality framework, our aim was to identify circumstances that predict formal care use among middle-aged and older adults in a setting where access is decoupled from an individual’s ability to pay for that care. To disentangle the effects of need, enabling and predisposing factors, and the offsetting effect of availability of informal care, we apply an intersectional framework and operationalise this by constructing regression trees for both formal and informal care.

## Methods

### Settings and participants

Data used were from the baseline (2010 to 2015) Canadian Longitudinal Study on Aging (CLSA), a random sample of 30,097 community-dwelling adults aged 45 to 85. Participants resided within a 25- to 50-km radius of 1 of the 11 data collection sites (Victoria, Vancouver, Surrey, Calgary, Winnipeg, Hamilton, Ottawa, Montreal, Sherbrooke, Halifax, St. John’s) in 7 Canadian provinces [33]. Those living in a First Nations community, an institution or a care facility, full-time members of the Canadian Armed Forces, anyone unable to speak French or English, or with cognitive impairment that made them unable to understand the study or answer basic personal questions were excluded.

### Sampling strategy and data collection

CLSA employs two sampling strategies for the Comprehensive Cohort used. Recruitment from provincial health registries (14% of the sample) followed random selection of eligible persons with 86% of the sample recruited through random digit dialing of landline telephone numbers for a given geographic area. After establishing eligibility of the respondent, consent was obtained either verbally or in writing. To ensure adequate representation of diverse demographic groups, the CLSA sample was stratified within each province by age group, sex, and distance from the data collection site [33]. Trained interviewers administered questionnaires in participants’ homes or a collection site. The CLSA reports a participation rate of 45% and an overall response rate of 10% [33].

### Assessment of formal and informal homecare use

Use of homecare was self-reported via answers to the following: "During the past 12 months, did you receive short-term or long-term professional assistance at home because of a health condition or limitation that affects your daily life, for any of the following activities?" and "During the past 12 months, did you receive short-term or long-term assistance from family, friends, or neighbours because of a health condition or limitation that affects your daily life, for any of the following activities?" Listed activities included personal care such as assistance with eating, dressing, bathing, or toileting, medical care such as help taking medicine or help with nursing care (for example, dressing changes or foot care), managing care such as making appointments, help with activities such as housework, home maintenance, or outdoor work, transportation, including trips to the doctor or for shopping and meal preparation or delivery.

### Potential predictors

#### Sociodemographic characteristics

Information used included sex, highest educational attainment (less than secondary school, secondary school graduation, some post-secondary, post-secondary graduation) and total personal income from all sources, before taxes and deductions, in the past 12 months as a categorical variable (<$20,000, $20,000-$49,999, $50,000-$99,999, $100,000-$149,999, ≥$150,000, Don’t know/No answer/Refused). By linking participants’ residence postal codes to dissemination areas, their place of residence was classified into ‘urban core’, ‘other urban’, and ‘rural’, a standard proxy measure for place of residence in Canada as per precedents [34]. Additional socio-demographic characteristics included age in years, country of birth, and time, in years, since immigration.

#### Family related variables

These included marital status (with partner, widowed, divorced, separated, single) and living arrangement (number of generations in the household) as they are previously documented family-related predictors of receiving formal and informal care at home [19] and in the community [15, 35].

#### Physical and mental health variables

To measure functional status, we used a composite variable derived by the CLSA that calculates the total number of times the respondent needed help with or was completely unable to do an activity in the Activities of Daily Living (ADL) and Instrumental Activities of Daily Living (IADL). This derived variable is a modification of the Duke Older Americans Resources and Services Multidimensional Assessment Questionnaire [36] that excludes questions regarding meal preparation ability. The final score (range 0–13) was then categorized into four groups: ‘0’ = *no activities of daily living problems*, ‘1–3’, ‘4–5’, and ‘more than 6’ as ‘*mild’*, *‘moderate’*, *and ‘severe’ activities of daily living problems*, respectively.

Perception of health was evaluated via two direct questions: “Would you say your health is excellent, very good, good, fair, poor?” and “Would you say your mental health is excellent, very good, good, fair, poor?” For analysis, we collapsed the first three categories into ‘good’ and the last two into ‘poor’ Self-Rated Health (SRH) and Self-Rated Mental Health (SRMH) categories. The 10-item version of the Centre for Epidemiologic Studies Depression (CESD) scale was used to measure depression with a cut-off of 10 or more indicating a positive screen for depression [37], and number of chronic conditions was entered into the analysis as a dichotomous variable (‘no self-report of chronic condition’ vs ‘at least one’).

#### Contextual variables

To measure contextual indicators of deprivation, Material and Social Derivation Indices (MSDI) for all Canadian census dissemination areas were retrieved from the ‘*Institut national de santé publique du Québec’* website and linked to participants’ postal codes [38]. Material indicators included 1) the proportion of the population aged 15 years and over with ‘education less than high school’, 2) with ‘no employment’ and 3) average income of the area. Social deprivation implies being separated, divorced or widowed, living alone, or in a single-parent family. The distribution of deprivation scores was then divided into quintiles with quintile 1 representing the population living in the most privileged area and quintile 5 living in the most deprived one.

### Ethics approval and consent to participate

The study was approved by the Queen’s University Health Sciences and Affiliated Teaching Hospitals Research Ethics Board (HSREB). Tools for Research at Queen’s (TRAQ) number: 6020994 in accordance with the ethical standards of the Declaration of Helsinki (1964) and its subsequent amendments. Informed consent was obtained by the CLSA from all participants before data collection.

### Statistical analysis

Descriptive analyses were performed to estimate the distributions of covariates, overall, and across the four care groups: 1) receiving formal care only, 2) receiving informal care only, 3) receiving a mix of formal and informal care, 4) receiving no care. Differences in distributions were compared statistically by Chi-square test for contingency tables.

To identify combinations of significant variables that predict care use we performed recursive partitioning regression tree analysis using Chi-Squared Automatic Interaction Detection (CHAID) analysis [39] as the growing method. Tree-Structured modeling techniques were developed more than four decades ago [40] mostly for data mining purposes, and are being used in health research with increasing frequency in recent years [41, 42]. CHAID is a classification method that examines the relative importance of each precursor in explaining the occurrence of a specified level of the outcome. It chooses the optimal partition based on χ2 calculated statistical significance adjusted using the Bonferroni correction. In order to allow more precursors in the models we set the tree depth limitation to five (instead of three levels which is the default for CHAID) and relaxed stopping criteria for splitting to a maximum size of 50 for parent nodes and of 20 for child nodes. This approach resulted in large trees with some nodes sized as small as 3. To generate more interpretable trees and meaningful nodes we then modified these criteria. In the final trees no parent node smaller than 100 was allowed to split and the minimum size for end nodes was set to 40. Applying this restriction decreased the final number of nodes of the formal care model from 41 to 35 and of informal care model from 38 to 36. To test trees’ stability and confirm the validity of the prediction accuracy (i.e., the correct classifications) of the models we ran a 10-fold cross-validation. This standard method of validation divides the sample randomly into 10 mutually exclusive subgroups, then each of the 10% folds serves once as a test sample while the rest of the sample (90%) is used to validate findings. The cross-validation risk, which is the proportion of cases incorrectly classified after adjustment for prior probabilities and misclassification, is the average of risk estimated across the 10 test samples. CHAID algorithm only prints the full-sample classification table. Separate algorithms were generated for ‘any informal care use’ and ‘any formal care use’ as the outcomes.

Analyses were performed using SAS version 9.4 (SAS Institute, Cary, NC, USA) and SPSS version 27.

## Results

Approximately half of those surveyed (50.9%) were female, 67% lived with a partner and more than 77% had postsecondary education. Overall, participants were high functioning: almost 90% reported no ADL or IADL limitation, or perceived their health as good (Table 1). Women received both formal and informal homecare more frequently (2.7% vs 1.9% among men for formal care and 10.1% vs 7.0% for informal care).

**Table 1:** Descriptive results by types of home care use.

Variable	Description	Total	Formal Only	Informal Only	Mixed Care	No care	P. value
**Sex**	Female	15308 (50.9)	409 (2.7)	1551 (10.1)	491 (3.2)	12857 (83.9)	<0.0001
	Male	14762 (49.1)	282 (1.9)	1033 (7.0)	277 (1.9)	13170 (89.1)	
**Age**	45–54	7592 (25.3)	76 (1.0)	636 (8.4)	109 (1.4)	6771 (89.2)	<0.0001
	55–64	9847 (32.8)	127 (1.3)	844 (8.6)	193 (2.0)	8683 (88.1)	
	65–74	7356 (24.5)	176 (2.4)	602 (8.2)	179 (2.4)	6399 (89.9)	
	75+	5275 (17.5)	312 (5.9)	502 (9.5)	287 (5.4)	4174 (79.0)	
**Marital status**	Single	2650 (8.8)	93 (3.5)	220 (8.3)	92 (3.5)	2245 (84.6)	<0.0001
	With partner	20638 (68.6)	309 (1.5)	1712 (8.3)	365 (1.8)	18252 (88.4)	
	Divorced/widowed/Separated	6774 (22.5)	289 (4.3)	652 (9.6)	311 (4.6)	5522 (81.4)	
**Education**	Less than secondary	1640 (5.5)	70 (4.3)	167 (10.2)	75 (4.6)	1328 (81.0)	<0.0001
	secondary graduation	2833 (9.4)	74 (2.6)	224 (7.9)	83 (2.9)	2452 (86.6)	
	Some post-secondary	2238 (7.4)	67 (3.0)	234 (10.5)	87 (3.9)	1850 (82.7)	
	Post-secondary degree/diploma	23309 (77.5)	477 (2.1)	1956 (8.4)	521 (2.2)	20355 (87.3)	
	Missing	50 (0.2)	3 (6.0)	3 (6.0)	2 (4.0)	42 (84.0)	
**Income**	Less than $20,000	4368 (14.5)	162 (3.7)	534 (12.2)	190 (4.4)	3482 (79.7)	<0.0001
	$20,000-$50,000	10529 35.0)	276 (2.6)	945 (9.0)	309 (2.9)	8999 (85.5)	
	$50,000-$100,000	9689 (32.2)	156 (1.6)	709 (7.3)	161 (1.7)	8663 (89.4)	
	$100,000-$150,000	2517 (8.4)	37 (1.5)	163 (6.5)	36 (1.4)	2281 (90.6)	
	$150,000 or more	1465 (4.9)	16 (1.1)	91 (6.2)	19 (1.3)	1339 (91.4)	
	Missing/refused	1502 (5.0)	44 (2.9)	142 (9.4)	53 (3.5)	1263 (84.1)	
**Country of born**	Canada	24620 (81.9)	582 (2.4)	2184 (8.9)	650 (2.6)	21204 (86.1)	<0.0103
	Other	5447 (18.1)	108 (2.0)	400 (7.3)	118 (2.2)	4821 (88.5)	
**Time since immigration**	≤10	254 (1.0)	3 (1.2)	9 (3.5)	0 (0)	242 (95.3)	<0.0001
	11–20	432 (2.0)	4 (1.0)	38 (8.8)	3 (1.0)	387 (89.6)	
	>20	4761 (15.8)	102 (2.1)	353 (7.4)	115 (2.4)	4191 (88.0)	
	Not-immigrant	24620 (81.2)	582 (2.4)	2184 (8.9)	650 (2.6)	21204 (86.1)	
**Living arrangement**	Single generation	20969 (69.7)	599 (2.9)	1834 (8.8)	609 (2.9)	17927 (85.5)	<0.0001
	Two generation	8526 (28.3)	76 (1.0)	693 (8.1)	139 (1.6)	7618 (89.4)	
	3 & more	508 (1.8)	11 (2.2)	49 (9.7)	17 (3.4)	431 (84.8)	
	Missing	67 (0.2)	5 (7.5)	8 (11.9)	3 (4.5)	51 (76.12)	
**Residence**	Rural	2422 (8.1)	36 (1.5)	207 (8.5)	55 (2.3)	2124 (87.7)	= 0.038
	Urban core	26059 (86.7)	611 (2.3)	2240 (8.6)	678 (2.6)	22530 (86.5)	
	Other urban	1211 (4.0)	29 (2.4)	94 (7.8)	23 (1.9)	1065 (87.9)	
	Missing	378 (1.3)	15 (4.0)	43 (11.4)	12 (3.2)	308 (81.5)	
**Physical limitation (ADL & IADL)**	No Problems	27037 (89.9)	368 (1.4)	1899 (7.0)	284 (1.1)	24486 (90.6)	<0.0001
	Mild problems	2558 (8.5)	261 (10.2)	552 (21.6)	319 (12.5)	1426 (55.8)	
	Moderate/severe	369 (1.2)	57 (15.4)	105 (28.3)	153 (41.5)	54 (14.6)	
	Missing	106 (0.4)	5 (4.7)	28 (26.4)	12 (11.3)	61 (57.6)	
**Depression**	No	25182 (83.7)	486 (1.9)	1953 (7.8)	491 (2.0)	22252 (88.4)	<0.0001
	Yes	4763 (15.8)	194 (4.1)	613 (12.9)	269 (5.7)	3687 (77.4)	
	Missing	125 (0.4)	11 (8.8)	18 (14.4)	8 (6.4)	88 (70.4)	
**Chronic condition**	None	2253 (7.5)	5 (0.2)	77 (3.4)	9 (0.4)	2162 (96.0)	<0.0001
	At least one	27606 (91.8)	679 (2.5)	2498 (9.1)	754 (2.7)	23675 (85.8)	
	Missing	211 (0.7)	7 (3.3)	9 (4.3)	5 (2.4)	190 (90.1)	
**SRH**	Good	27272 (90.7)	526 (1.9)	2037 (7.5)	495 (1.8)	24214 (88.8)	<0.0001
	Poor	2776 (9.2)	164 (5.9)	543 (19.6)	272 (9.8)	1797 (64.7)	
	Missing	22 (0.1)	1 (4.6)	4 (18.2)	1 (4.6)	16 (72.7)	
**SRMH**	Good	28395 (943.4)	621 (2.2)	2301 (8.1)	653 (2.3)	24820 (87.4)	<0.0001
	Poor	1650 (5.5)	68 (4.1)	282 (17.1)	114 (6.9)	1186 (71.9)	
	Missing	25 (0.1)	2 (8.0)	1 (4.0)	1 (4.0)	21 (84.0)	
**Material Deprivation Score Quintile within Province**	1 (lowest)	10842 (36.1)	235 (2.2)	891 (8.2)	251 (2.3)	9465 (87.3)	= 0.0253
	2	7479 (24.9)	157 (2.1)	648 (8.7)	185 (2.5)	6489 (86.8)	
	3	4915 (16.4)	111 (2.3)	451 (9.2)	126 (2.6)	4227 (86.0)	
	4	3447 (11.5)	81 (2.4)	297 (8.6)	102 (3.0)	2967 (86.1)	
	5 (Highest)	2236 (7.4)	71 (3.2)	196 (8.8)	67 (3.0)	1902 (85.1)	
	Missing	1151 (3.8)	36 (3.1)	101 (8.8)	37 (3.2)	977 (84.9)	
**Social Deprivation Score Quintile within Province**	1 (lowest)	5279 (17.6)	79 (1.5)	363 (6.9)	93 (1.8)	4744 (89.9)	<0.0001
	2	5512 (18.3)	103 (1.9)	440 (8.0)	97 (1.8)	4872 (88.4)	
	3	5187 (17.3)	112 (2.2)	434 (8.4)	114 (2.2)	4527 (87.3)	
	4	6028 (20.1)	144 (2.4)	575 (9.5)	179 (3.0)	5130 (85.1)	
	5 (Highest)	6918 (23.0)	217 (3.1)	671 (9.7)	248 (3.6)	5777 (83.6)	
	Missing	1151 (3.8)	36 (3.1)	101 (8.8)	37 (3.2)	977 (84.9)	

*Note*: Values inside parentheses are row percent; P.values are from Chi-square tests.

Need, as indicated by limitations in ADL/IADL, was the primary determinant for receiving care, whether formal or informal; using regression trees language, need was the first ‘splitter’ or branch of the tree. The next level of possible predictor was different for formal and informal care. We, therefore, report detailed results separately for formal and informal care outcomes.

### Receiving formal care

By generating a total of 35 nodes and 19 terminal nodes CHAID identified that 28,611 (95.1%) participants did not receive formal care and 1,459 (4.9%) did. The overall risk of misclassification was 4.5% and remained almost the same (4.6%) after 10-fold cross-validation.

**Fig 1:**
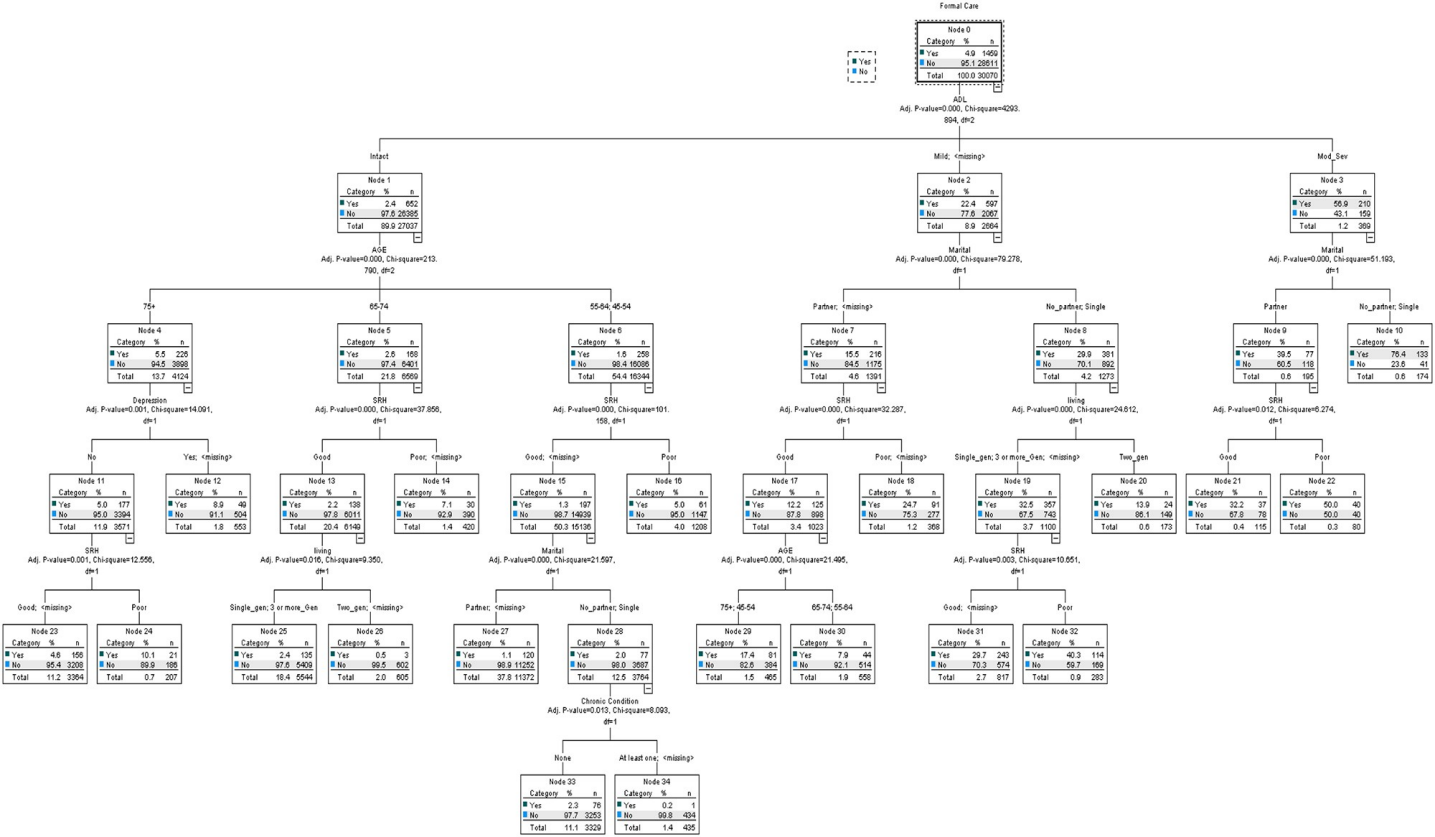
Regression tree: Use of formal home care.

As stated above, the primary precursor or possible predictor (the first splitter) of formal care use was limitation in ADL and IADL. Among those with moderate to severe functional limitations 57% received formal care. The comparable proportion among those with mild limitation was 22.4%. For those with any level of ADL limitation the next ‘branch’ in the tree, that is, the next indicator was having no partner. Among the moderate to severely limited group, 9.5% of those with a partner received formal care compared with 76.4% of those with no partner. Comparable proportions for those with mild limitation were 15.5% and 29.9%, respectively. Further dissecting the branches of the tree to find intersecting characteristics that aligned with receiving care (i.e. beyond partner status) identified subsequent possible predictors for formal care to be: SRH (for moderate/severe ADL limitation + partner: 32.2% with good SRH received care vs 50.0% in the poor SRH group), living arrangement + SRH for the mild limitation with no partner group, and SRH + age for mild limitation with partner group (Fig 1).

Among those with no functional limitations (89.9%), 2.4% received formal care. This allocation of formal care was predicted best by age (45–64 Y– 1.6%, 65–74 Y– 2.6%, 75+ Y– 5.5%). For those younger than age 75, SRH and family-related variables such as partner status and living arrangement were important, whereas in the older than 75 group, self-reported depression also played a role.

Overall, sex, that is, being a man or a woman, was not a branch point for formal care. Of particular note measures of SES such as income or education also did not have any impact on receiving formal care.

### Informal care

**Fig 2:**
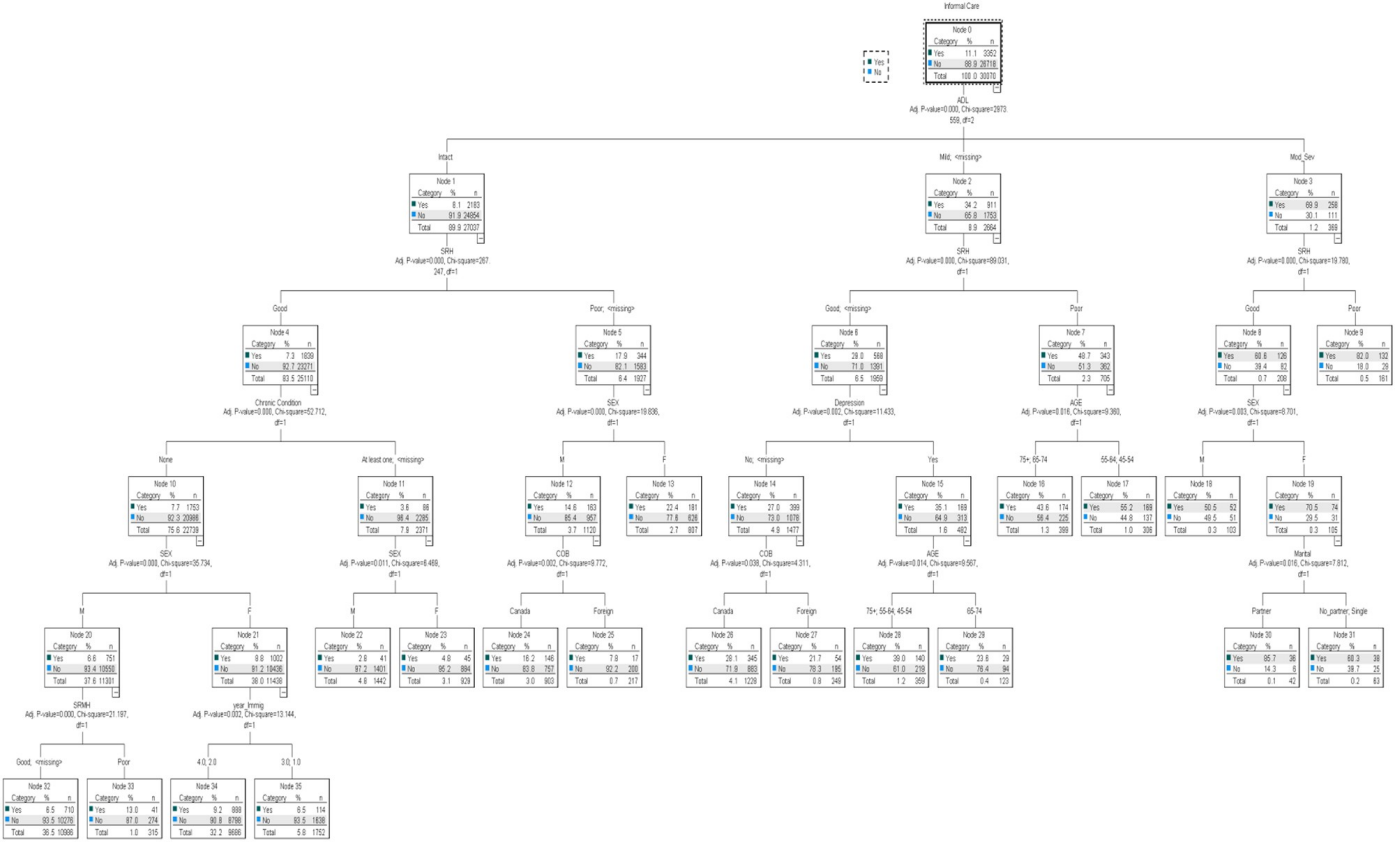
Regression tree: Use of informal home care.

As with formal care, the main precursors of informal care, were ADL/IADL limitations, SRH, partner status and depression. In contrast to receiving formal care, however, sex, and immigration status also emerged as important (Fig 2). With moderate to severe ADL limitations, partner status remained important but only among women. In this group almost 86% of women with a partner received informal care compared with only 60% of women with no partner. Age was a possible predictor of receiving informal care regardless of SRH status in those with mild functional limitations. Country of birth was predictive for those with no depression symptoms. Canadian-born participants were somewhat more likely to receive informal care than were foreign-born individuals (28% vs 22%).

The pattern of receiving informal care was more complex among those (7.3%) with no ADL limitations, showing intersections among sex, immigration status and health. Country of birth aligned with informal care use for men with poor SRH, but not for women. In those with good SRH and no chronic condition years since immigration predicted informal care use for women.

The regression tree generated by the CHAID algorithm for informal care use had almost twice the risk of misclassification relative to the tree for formal care use. The final tree, which included a total of 36 nodes and 19 terminal nodes, correctly classified 26,470 (99.1%) of those who did not receive informal care and 427 (12.7%) of those who received this level of care. Overall risk of misclassification, therefore, was calculated at 10.6%. The risk increased slightly to 10.8% after 10-fold cross-validation.

## Discussion

While need, as measured by ADL/IADL, was the most important antecedent of both formal and informal care use, other possible precursors varied considerably across the two types of care. This is consistent with theoretical conceptualisations that consider formal and informal care use as different entities [9, 43]. In contrast to findings that socio-economic status (SES) measured in various ways predicted formal care use in other countries [15, 16, 44], in the Canadian context we have studied this did not emerge. While variability in SES definitely exists in Canada, Medicare seems to ameliorate SES related inequalities in home care utilisation. With government funding for essential although not always optimal formal homecare, previously identified barriers of sex or SES are no longer apparent as sources of inequity. Those whose lives were marked by the isolation of having no partner or living alone inevitably had less access to informal care, but this was offset by greater use of formal care. We cannot, however, determine whether living with someone rather than alone acted as a barrier to receiving formal care. Although individual circumstances are not amenable to change via social policies, perhaps in a setting such as Canada, where, without financial penalty, formal care can offset a lack of informal care, government funded formal care may minimise the harms of socioeconomic or individual deficits.

Our findings diverge somewhat from those of Gilmour [4] whose multivariate analysis of formal home care use among Canadians demonstrated that those of lower socioeconomic status received more care. There are several possible explanations for this divergence. Although the timeframe studied was similar, Gilmour included populations age 12+ whereas our study was limited to adults aged 45+. Perhaps more important is that multivariate analyses [4] examine the impact of single characteristics while controlling for all others, while recursive partitioning regression tree analysis (our research design) begins to tease out intersections of various characteristics. This has the potential to enrich findings. Both papers do demonstrate that with government funding for home care, the disadvantages of economic deprivation disappear.

We examined whether precursors of care use changed with different levels of ADL/IADL limitation. Starting from need and dividing the study population into those who did and did not have limitations the next characteristics that aligned with any care use were marital status, SRH, age, number of chronic conditions, and depression. We explored whether intersections among these factors explained variability in care use. Of note, SES measured either at the individual level by income and education, or at a contextual level by deprivation indices and residence location was not of significance. Sex, country of birth, and years since immigration were aligned with informal but not formal care. Family supports were, however, important sources of care for those with no ADL limitations. Informal care use was also a function of an individual’s immigration status. These findings are consistent with existing precedents that consider informal and formal care receiving as different ‘care’ behaviours [9].

Results support the construct that deep patterns of care use will be uncovered when an intersectional approach is adopted. Utilizing recursive partitioning methods revealed complex interactions for high risk subpopulations at specific, intersecting social locations. We were able to identify factors that intersect as possible predictors of formal and informal care use. Of note, in the Canadian context of government funded formal care, sex was not a predictive characteristic for access to it, while living arrangement was. However, as living arrangements in old age are profoundly gendered (men are more likely to live with spouses while women live alone), the finding that both formal and informal care depended on living arrangements, raises relevant gender issues [45]. Older men are more likely to provide informal care to their spouses [46–48], adding to the interplay of the social factor, gender, in shaping care use patterns in old age. Our exploration of intersecting circumstances demonstrates that it was not sex, alone, that predicted care utilisation but rather the effect of living alone, a reality faced disproportionately by older women [49].

Our regression tree models hinted that potential unmet care needs remain. For example, 43% of individuals with moderate to severe ADL limitations did not receive formal homecare. This might be explained by marital status and associated availability of informal care, as those with partners were the ones less likely to receive formal care (40% vs 76% in those with no partner). Among participants with no ADL limitations 562 (2.4%) received formal and 2183 (8.1%) received informal care. ADL limitation, although a key precursor of care use, is not the sole medical or socio-demographic reason adults might seek care. We identified other possible and plausible antecedents such as living arrangement, age, and SRH in participants with no ADL limitation, however, results of recursive partitioning remain exploratory and should be confirmed via future etiological analyses.

### Methodologic strengths of this study

The methods used are novel and have allowed for a deep ‘dissection’ of intersecting characteristics that predict use of formal care, and revealed how social safety nets such as Medicare can correct for previously reported inequities. Ordinary regression models are focused on the estimation of risk in high-risk groups. Identification of risk factors in lower-risk populations (for example in those with no or only mild ADL limitations) is an advantage of recursive partitioning models. By recursive and stepwise subgroupings, combinations of these subgroups can be interpreted as social locations and hence provide a *quantification* of intersectionality [23]. Within-group heterogeneity usually is overlooked when epidemiological studies use traditional analytic approaches, while regression trees reveal the sources of this heterogeneity. Most existing studies with similar research questions have explored data via regression analyses [23, 50]. The few that used regression trees [14, 41, 51] did not conceptualise determinants of care use based on intersectionality theory and therefore did not find the complex interplay between potential associated factors. We have demonstrated the shortcomings of relying on diminished function (e.g limitations in ADL/IADL) as the sole indicator of need for care and have identified factors (for example age and living arrangement in those with no ADL limitations) that predict care use in higher functioning individuals. Both models, constructed following the CHAID algorithm, produced stable trees after 10-fold cross-validation with 4.6% risk of misclassification for formal care use and 10.7% risk of misclassification for informal care use.

### Limitations

The CLSA follows a very robust sampling strategy and is representative of middle-aged and older Canadians living in the community. Our aim was to identify social and individual characteristics aligned with use of homecare and, hence, exclusion of those living in institutions seems like a minor issue. Institutional care, however, may act as a replacement for homecare, especially for older adults who live alone and have significant needs. We cannot determine whether this exclusion had any impact on our results. The survey data available does not differentiate those whose formal care is state funded from those paying privately for such care. However, Canadian data cited above show that 91% of nursing care (the key component of what we have defined as formal care) required no funding from the recipient, suggesting that although private funding sometimes pays for home support (e.g. cleaning, food preparation, etc) it rarely is needed to cover medical care [4]. Other excluded groups such as those living in First Nations communities, or those unable to speak French or English, may represent different care needs and use behaviours that were not captured in the dataset. Homecare use variables were self-reported and binary rather than quantified, and do not distinguish between, for example, occasional or almost full-time care. The data used for this study were collected prior to the COVID pandemic. Its impact on labour supply, availability of formal care and access to it may have shifted in ways that are as yet to be identified. Finally, the results of this study can only be interpreted as exploratory. Cross-sectional data cannot identify causality or the direction of any associations found. Confirmatory analyses using longitudinal data and possibly causal mediation techniques remain to be undertaken.

## Conclusions

Using a novel methodology we found that the impact of inequities associated with social locations or individual isolation on use of formal homecare diminishes or even disappears when government-funded medical care is universally available. Nevertheless, identification of vulnerable subgroups, both those who did and those who did not receive care, highlights subpopulations that might benefit from increased clinical attention or public health interventions. Future research might explore the interplay between formal and informal homecare in settings with varying levels of state funded formal care. With aging of populations throughout the world, a deeper understanding of care use patterns will be essential for designing delivery systems that are not wasteful and that ensure access to high-quality care for all who need it.

## Abbreviations

ADL: Activities of Daily Living
CESD: Centre for Epidemiologic Studies Depression
CHAID: Chi-Squared automatic interaction detection
CLSA: Canadian Longitudinal Study on Aging
IADL: Instrumental Activities of Daily Living
SES: Socio-economic status
SRH: Self-rated health
SRMH: Self-Rated Mental Health
